# Can patient decision aids reduce decisional conflict in a de-escalation of breast radiotherapy clinical trial? The PRIMETIME Study Within a Trial implemented using a cluster stepped-wedge trial design

**DOI:** 10.1186/s13063-021-05345-y

**Published:** 2021-06-14

**Authors:** Indrani S. Bhattacharya, Joanne S. Haviland, Lesley Turner, Hilary Stobart, Ada Balasopoulou, Liba Stones, Anna M. Kirby, Cliona C. Kirwan, Charlotte E. Coles, Judith M. Bliss, on behalf of the PRIMETIME Trialists

**Affiliations:** 1 The Institute of Cancer Research Clinical Trials and Statistics Unit (ICR-CTSU), London, UK; 2 Oncology & Radiotherapy, Addenbrooke’s Hospital, Cambridge University Hospitals NHS Foundation Trust, Cambridge, UK; 3 Independent Cancer Patients’ Voice, London, UK; 4 Royal Marsden NHS Foundation Trust, London, UK; 5 Institute of Cancer Research, London, UK; 6 Institute of Cancer Sciences, University of Manchester, Manchester University NHS Foundation Trust, Manchester, UK; 7 Department of Oncology, University of Cambridge, Cambridge, UK

**Keywords:** Breast, Cancer, Decisional-conflict, De-escalation, Oncology, Radiotherapy, Cluster, Stepped-wedge, SWAT

## Abstract

**Background:**

For patients with early breast cancer considered at very-low risk of local relapse, risks of radiotherapy may outweigh the benefits. Decisions regarding treatment omission can lead to patient uncertainty (decisional conflict), which may be lessened with patient decision aids (PDA). PRIMETIME (ISRCTN 41579286) is a UK-led biomarker-directed study evaluating omission of adjuvant radiotherapy in breast cancer; an embedded Study Within A Trial (SWAT) investigated whether PDA reduces decisional conflict using a cluster stepped-wedge trial design.

**Methods:**

PDA diagrams and a video explaining risks and benefits of radiotherapy were developed in close collaboration between patient advocates and PRIMETIME trialists. The SWAT used a cluster stepped-wedge trial design, where each cluster represented the radiotherapy centre and referring peripheral centres. All clusters began in the *standard* information group (patient information and diagrams) and were randomised to cross-over to the *enhanced* information group (standard information plus video) at 2, 4 or 6 months. Primary endpoint was the decisional conflict scale (0–100, higher scores indicating greater conflict) which was assessed on an individual participant level. Multilevel mixed effects models used a random effect for cluster and a fixed effect for each step to adjust for calendar time and clustering. Robust standard errors were also adjusted for the clustering effect.

**Results:**

Five hundred twenty-one evaluable questionnaires were returned from 809 eligible patients (64%) in 24 clusters between April 2018 and October 2019. Mean decisional conflict scores in the *standard* group (N = 184) were 10.88 (SD 11.82) and 8.99 (SD 11.82) in the *enhanced* group (N = 337), with no statistically significant difference [mean difference − 1.78, 95%CI − 3.82–0.25, p = 0.09]. Compliance with patient information and diagrams was high in both groups although in the enhanced group only 121/337 (36%) reported watching the video.

**Conclusion:**

The low levels of decisional conflict in PRIMETIME are reassuring and may reflect the high-quality information provision, such that not everyone required the video. This reinforces the importance of working with patients as partners in clinical trials especially in the development of patient-centred information and decision aids.

**Supplementary Information:**

The online version contains supplementary material available at 10.1186/s13063-021-05345-y.

## Introduction

Adjuvant radiotherapy following breast conserving surgery (BCS) plays an important role in the treatment of early breast cancer. The absolute benefit of radiotherapy is dependent on the individual patient’s prognosis. Radiotherapy carries risk. For some patients with very low-risk of local relapse, radiotherapy risks may outweigh the benefits, and for these patients, risk adaptation of treatment to omit radiotherapy may be preferable, with this hypothesis being under evaluation in several studies [1–5]. Treatment de-escalation may increase patient uncertainty (decisional conflict) in relation to their care pathway. Uncertainty may be increased if insufficient information is provided. Supplementing standard patient information material with patient decision aids (PDA) has been hypothesised to reduce decisional conflict. PDA are tools helping patients understand treatment risks and benefits, consider values placed on the risk-benefit ratio and participate with clinicians in deciding treatment options. Testing of the hypothesis that PDA reduce decisional conflict requires evaluation in the context of a clinical trial, for example via a ‘Study Within A Trial’ (SWAT). A SWAT is a research study embedded within a clinical trial enabling assessment of different ways of designing, conducting, analysing and evaluating components of the research conduct [6].

PRIMETIME is a UK-led biomarker-directed interventional cohort study aiming to identify a group of breast cancer patients who can safely avoid adjuvant radiotherapy following BCS [1]. A SWAT was conducted within PRIMETIME to identify whether PDA reduced decisional conflict in patients considering treatment de-escalation. In this paper we report the development of the PRIMETIME PDA and SWAT execution.

## Methods

### Context for SWAT

Details of PRIMETIME have been published previously [1]. The biomarker IHC4+C (incorporating Ki-67) is used to determine the patient’s recurrence risk [7]. Patients predicted to be at very-low risk are directed to avoid radiotherapy, and patients at low, intermediate or high-risk are directed to receive radiotherapy as per standard of care [1]. Patients both accepting and declining the recommendation are followed up. Patients were able to consent to the SWAT even if they subsequently declined PRIMETIME.

### PDA development

**Fig. 1:**
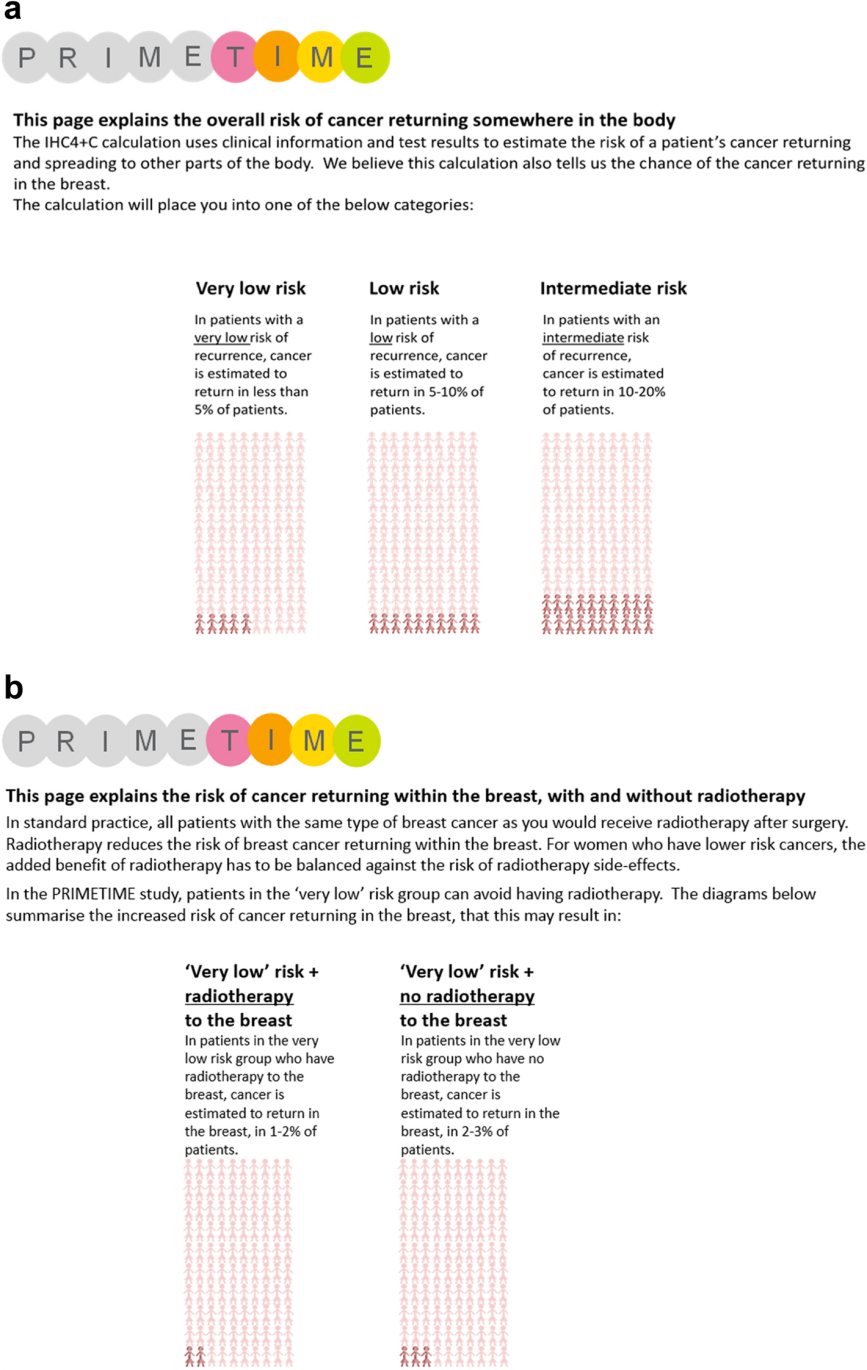
**a** Patient information diagrams explaining the risk of distant recurrence in patients at very low, low and intermediate risk. **b** Patient information diagrams explaining the risk of local recurrence in patients who do and do not receive radiotherapy in the very low risk group

PDA were developed in close collaboration with PRIMETIME patient advocates and designed to be used in conjunction with patient information sheets. Diagrams were designed to explain the risks and benefits of radiotherapy using natural frequency formats (numerical values expressed as event rates in groups with and without the intervention). They also explained the risks of recurrence in the different risk groups and compared recurrence risk in patients receiving and not receiving radiotherapy in the low-risk group (Fig. 1a, b). Diagrams were designed to be used in the clinic consultation with the healthcare professional and patient present.

Building on the written information it was considered that radiotherapy risks and benefits could also be presented in a different format such as a video to be watched by patients independently. A working group consisting of patient advocates and PRIMETIME Trial Management Group members, together with a series of patient focus groups established content to be included in the PDA video. The PDA were designed according to criteria outlined by the International Patient Decision Aid Standards [8].

### SWAT development and execution

All patients being approached for PRIMETIME were eligible for the SWAT; all sites participated. The SWAT was implemented using a cluster stepped-wedge trial design. The stepped-wedge design consists of the sequential implementation of an intervention to participants grouped within clusters over a number of time periods [9]. Cluster randomisation ensured all patients within a single site received uniform information for specified time periods. The stepped-wedge design enabled all site clusters to receive the intervention sooner than in a parallel-group cluster randomised trial, where all clusters could be given access to the intervention at the end of the study. Each site cluster was defined as the radiotherapy centre and its non-treating referral sites. All sites began in the *standard* group which included patient information sheet and diagrams; at pre-specified time-points, site clusters switched to the *enhanced* group, which included patient information sheet, diagrams, and video. The intervention (video) pertained to both the cluster and individual participant level.

After patients decided whether or not they wished to participate in PRIMETIME, patients were asked to complete a questionnaire (Appendix figure 1 and 2). Questionnaires assessed decisional conflict using a validated tool and patients were asked to indicate their highest level of education. Of note, the outcome was assessed at individual patient level. Questionnaires (paper-based) were distributed to patients in the clinic. Return of the questionnaire indicated patient consent to the sub-study.

Site clusters were allocated, via minimisation, to switch from *standard* to *enhanced* information at 2, 4 or 6 months. Minimisation was performed manually using a single balancing factor of prior recruitment to the IMPORT HIGH [10] and or FAST FORWARD [11] breast radiotherapy trials, as an indication of trial research experience. Minimisation was performed at the Institute of Cancer Research Clinical Trials and Statistics Unit. Each site cluster was informed of their cross-over date via email after their first patient had consented to the SWAT. Access to the video was restricted until 1 week before cross-over at which point an email containing a web link to the video and DVDs were sent to the centres for patients without internet access.

The SWAT primary endpoint was the decisional conflict score (0–100, with greater scores indicating more decisional conflict) [12] which was assessed on an individual participant level. Secondary endpoints were acceptance of entry into PRIMETIME and acceptance of the recommended treatment within PRIMETIME. The decisional conflict subscale scores of uncertainty, informed, values clarity, support and effective decision were also assessed.

### Statistical methods

The SWAT target sample size was 264 patients based on three steps in the cluster stepped-wedge trial design (at 2, 4 and 6 months) of 33 site clusters (11 per step), with 2 patients/site cluster/2-month period. Of note, the number of participants within each cluster and number of clusters was unknown as the SWAT was planned within a newly recruiting trial. Equal cluster sizes were assumed. There is no published definition of a clinically significant reduction in decisional conflict; two studies conducted in similar populations to PRIMETIME found effect sizes around 0.40, with standard deviations for the total decisional conflict scale score ranging from 11 to 25 [13, 14]. There are no published data on the intraclass correlation (ICC) for the decisional conflict scale. Assuming α = 0.05, 264 patients from 33 site clusters would have ≥ 80% power across the full range of ICC values (0–1) to detect a 10-point difference in total score for the decisional conflict scale (effect size = 0.55, standard deviation = 18). Recruitment was extended beyond the original accrual target until all site clusters had switched as per protocol with cluster randomised stepped-wedge trials.

Analyses were conducted on an intention-to-treat basis, with questionnaires analysed according to the cross-over date regardless of whether patients reported having watched the video. Of note, each patient completed a single questionnaire. Multilevel mixed effects models used a random effect for cluster and a fixed effect for each step to adjust for calendar time and clustering. Robust standard errors were also adjusted for the clustering effect [15, 16]. A linear regression model was used for the decisional conflict scale and subscales; an estimate of the difference in mean scores pre- and post- video-implementation was obtained (with 95% confidence interval, CI), and groups were compared using the z-test. Secondary endpoints (acceptance of entry into PRIMETIME and recommended treatment) used logistic regression and were reported as odds ratios (OR) with 95%CI. Additionally, total decisional conflict scores were dichotomised using a cut-off of ≥ 25 to define ‘clinically significant’ decisional conflict [17, 18], and groups were compared using logistic regression as for the secondary endpoints. Exploratory analyses including age and education in the models assessed associations with decisional conflict. The ICC value for the overall decisional conflict score was estimated from the primary endpoint model.

There is no published guidance for dealing with missing data in the decisional conflict scale, and so EORTC guidance for quality of life measures was used [19], whereby missing items are imputed from the mean of completed items providing ≥ 50% of the questions are completed.

Analyses used Stata version 14 based on a data snapshot taken on 25 October 2019. The SWAT was approved by the East of England Research Ethics Committee on 22nd February 2018 (16/EE/0305). PRIMETIME (ISRCTN 41579286) is funded by Cancer Research UK (C17918/A20015). The SWAT was registered in the SWAT store - MRC Hubs for Trials Methodology Research (SWAT 56) [20].

## Results

### PDA video development

Focus groups determined the video should build on the existing patient information sheets and diagrams, providing the same information but in a different format. Patient advocates felt providing additional information would not only be overwhelming, but unethical to have differing content available to participants. Specific themes from existing materials the advocates advised highlighting in the video included; risks of recurrence, benefits and side effects of radiotherapy and lack of clear survival benefit from radiotherapy for low-risk breast cancer. Also, the possibility of treating any subsequent local recurrences radically with surgery +/− radiotherapy was highlighted. It was also felt important to highlight that patients not receiving radiotherapy would undergo extra mammograms from years 6 to 10 and therefore be monitored more intensively compared with standard of care.

A script was developed using a question-based format, including an explanation of why the PRIMETIME study was being run, what was needed to calculate the patient’s risk and how we weigh up the risks and benefits of radiotherapy (Appendix table 1). The video was developed in collaboration with Eyewitness productions, who also produced the interactive graphics explaining recurrence risk based on the diagrams (Appendix figure 3). The side effects of radiotherapy were explained similarly (Appendix figure 4). The video is available at https://www.icr.ac.uk/primetime.

### SWAT execution

**Fig. 2:**
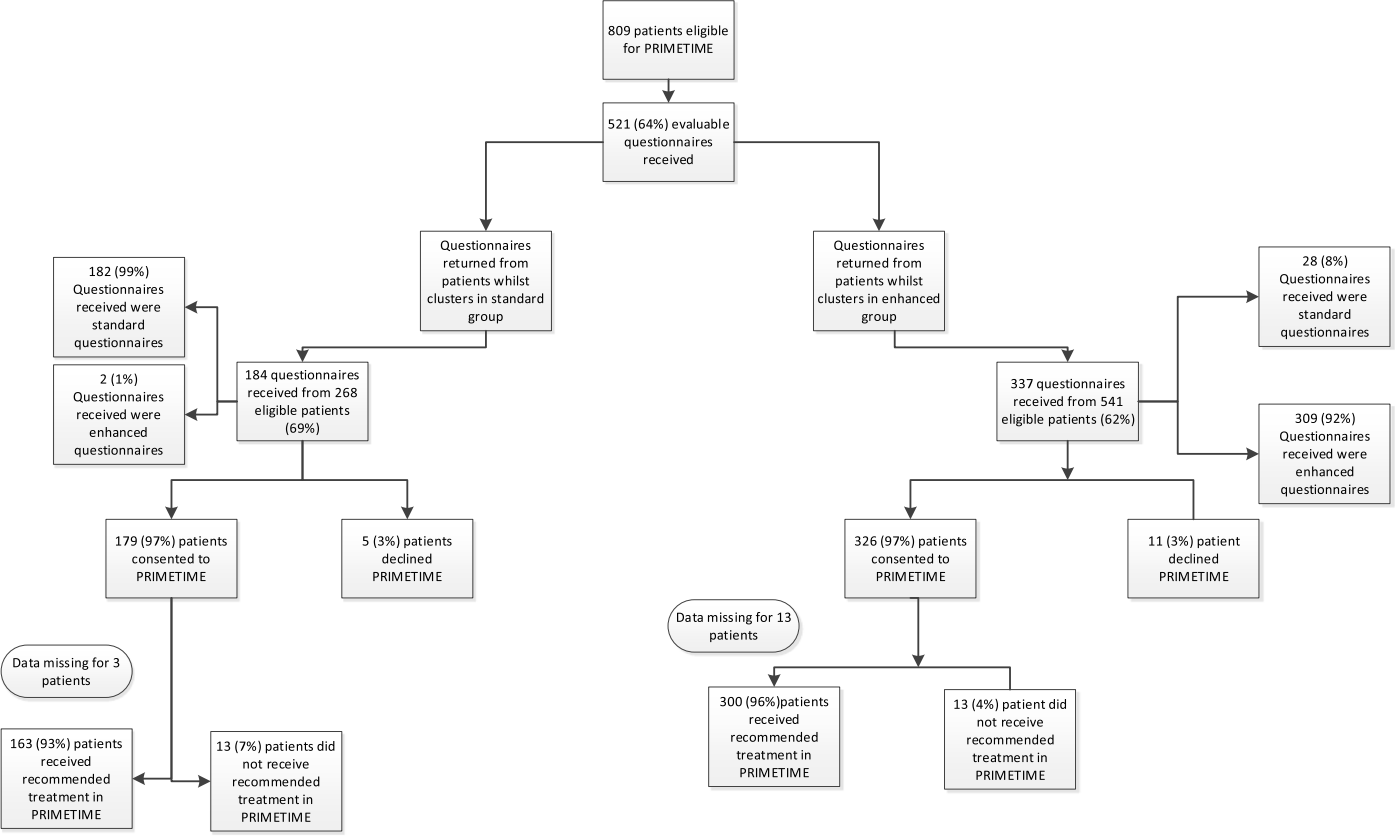
Consort—‘participant flow diagram’

Five hundred twenty-one evaluable questionnaires were returned from 809 eligible patients (64% return rate) [Fig. 2] in 24 clusters (Table 1) between April 2018 and October 2019. Median ages (interquartile range) of those patients who did and did not consent to the SWAT were 69 (65–72) and 68 (64–72) respectively. With regard to questionnaire return, 184 questionnaires were returned by the *standard* group and 337 returned by the *enhanced* group. Median age was similar between the *standard* and *enhanced* groups [70 versus 68 years respectively], as was education level (Table 2). There were no differences in distribution of age or education level over the time period of the study. All patients in both groups read the patient information sheet. However, compliance with the additional material varied; of those with available data, 135 (73%) and 290 (86%) reported using the diagrams in the *standard* and *enhanced* groups respectively. In the enhanced group, 121 (36%) reported watching the video, 172 (51%) did not and 44 (13%) had missing data. There were no differences in age and education level between those who did and did not watch the video.

**Table 1:**
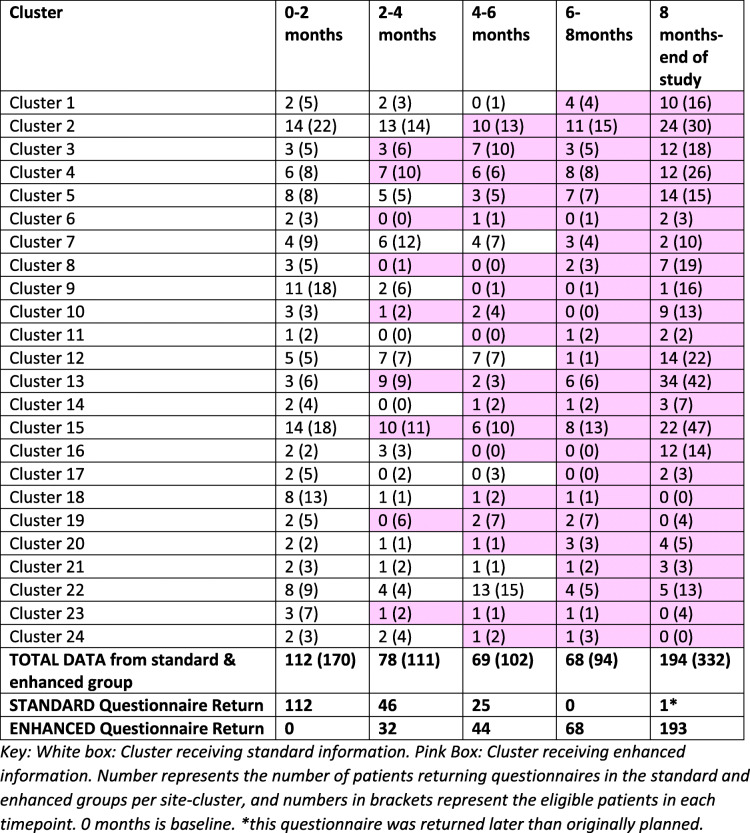
Summary of questionnaires returned from eligible patients per site cluster in the standard and enhanced groups in the PRIMETIME SWAT *(eligible patients in brackets)*

White box: Cluster receiving standard information. Pink box: Cluster receiving enhanced information. Number represents the number of patients returning questionnaires in the standard and enhanced groups per site cluster, and numbers in brackets represent the eligible patients in each timepoint. 0 months is baseline. *This questionnaire was returned later than originally planned

**Table 2:** Summary of baseline characteristics and information use in patients in the standard and enhanced groups

	Standard group ***N*** = 184 (%)	Enhanced group ***N*** = 337 (%)
Age (median, IQR)	70 (66-73)	68 (65-72)
Age categories:		
60–64	40 (22)	77 (23)
65–69	52 (28)	121 (36)
70–74	55 (30)	97 (29)
≥ 75	37 (20)	42 (12)
Education level*		
PG degree/degree	43 (24)	83 (27)
A-level/HND	28 (15)	45 (14)
School cert/O-level	59 (32)	118 (38)
No formal education	50 (27)	66 (21)
Patients reading PIS		
Yes	176 (96)	331 (98)
No	0	0
Missing	8 (4)	6 (2)
Patients looking at		
diagrams	135 (73)	290 (86)
Yes	35 (19)	38 (11)
No	14 (8)	9 (3)
Missing		
Patients watching video	N/A	
Yes		121 (36)
No		172 (51)
Missing		44 (13)

*Data regarding education level missing for 4 patients in standard group and 25 patients in the enhanced group. Percentages calculated using all available data

Mean decisional conflict scores in the *standard* group (N = 184) were 10.88 (SD 11.82) and 8.99 (SD 11.82) in the *enhanced* group (N = 337). There was no statistically significant difference in decisional conflict scores between the groups [estimated mean difference − 1.78, 95% CI − 3.82–0.25, p = 0.09; effect size 0.08]. A negative value indicates a reduction in mean scores in the *enhanced* compared with the *standard* group. There were no apparent differences between decisional conflict across time between the two groups.

Clinically significant decisional conflict was reported in fewer patients in the *enhanced* group (62/377, 16%) compared with 42/184 (23%) in the *standard* group, although this difference was not statistically significant [OR 0.67 (0.40–1.11), p = 0.12].

The majority of patients who returned questionnaires in the SWAT had consented to PRIMETIME [179/184 (97%) and 326/337 (97%) patients in the *standard* and *enhanced* groups respectively] (Fig. 2), with no statistically significant difference between groups [OR = 0.95 (0.17–5.22), p = 0.95]. For patients with available data, 163 (93%) patients in the *standard* group opted for their recommended treatment and 13 (7%) did not; in the *enhanced* group, 300 (96%) patients opted for their recommended treatment and 13 (4%) did not [Fig. 2]. There was no statistically significant difference in patients accepting the recommended treatment in PRIMETIME according to whether they were in the *standard* or *enhanced* groups [OR = 1.17 (0.59–2.29), p = 0.66].

There was no significant association between either age or education level and decisional conflict scores when allowing for the effects of the *standard* and *enhanced* groups and time to cross-over (Appendix table 2). There were also no significant differences in subscale scores between the groups (Appendix table 3). The ICC for total decisional conflict score was estimated to be 0.03.

## Discussion

PDA were designed in close collaboration with patient advocates to help patients consider the risks and benefits of adjuvant breast radiotherapy. The PRIMETIME SWAT investigated whether the addition of a video to patient information sheets and diagrams could reduce decisional conflict. We found that absolute levels of decisional conflict were low on average in both the standard and enhanced groups, with no significant reduction in decisional conflict following video implementation and that less than half of the patients reported watching the video. There was no statistically significant difference in the acceptance of PRIMETIME entry or recommended treatment between the two groups.

Development of the PDA was primarily patient-led with advocates identifying important concepts in breast cancer radiotherapy which needed to be communicated to patients. This was facilitated by a series of focus groups where patients and healthcare professionals established the most important concepts for patients to understand when considering a de-escalation trial such as PRIMETIME. These concepts fed into the comprehensive PDA development process. Patient advocates also identified as a challenge being able to clearly explain concepts such as risks of recurrence. In general, patients may be able to more accurately perceive risk when numerical values are used. Using natural frequency formats and expressing probabilities as an event rate out of 100 or 1000 patients can help improve understanding [21]. Natural frequency formats were therefore used throughout the PRIMETIME PDA to aid patient understanding. PDA were designed relatively simply and cheaply to be easily usable for patients both within and independent of the clinic consultation. Although the SWAT preceded the COVID-19 pandemic, there has now been an acceleration into a practice of fewer face-to-face consultations, increase in telephone/video consultations and remote consent. This makes the use of PDA and videos particularly timely to help patients and clinicians in the informed consent process.

The PDA were tested using the SWAT concept embedded within the PRIMETIME study across UK cancer centres. This enabled questions regarding decisional conflict in this population to be answered in parallel with the primary question of the main trial which was to identify a group of patients with low-risk breast cancer who can safely avoid radiotherapy. However, it was found the response rate for this SWAT was only 64%. Of note, there were no significant differences in age between those patients who consented to or declined the SWAT (age was the only baseline characteristic available for comparison). An important consideration is that this SWAT encompassed a broader patient group than that entered into the main trial, including those who declined entry into the main trial. Although trial guidance was that all patients who were eligible for PRIMETIME were to be offered entry to the SWAT, it may have been that sites were not able to offer the SWAT for example due to capacity issues. Patients declining the SWAT may have different characteristics or levels of decisional conflict. It is therefore important that sites are supported to approach these patients so they are given the opportunity to participate in other studies albeit with a separate consent process. Also, it was found that data were missing within the SWAT including whether patients watched the video. Missing data can be a challenge in trials but possibly even more so in a SWAT.

Regarding the numbers of questionnaires returned in each group, 184 questionnaires were returned from 268 eligible patients (69%) and 337 questionnaires were returned from 541 eligible patients (62%), in the standard and enhanced groups respectively. The reason for the greater number of questionnaires returned in the enhanced group may be explained by SWAT recruitment improving over time meaning that recruitment may coincidentally improve whilst sites were in the enhanced group. It should also be noted that a requirement of a stepped-wedge trial is that all sites must remain open until every site has crossed over. This may result in some sites spending extended periods of time in the enhanced group. The mean decisional conflict scores were not statistically significantly different between the groups, although the proportion of patients with ‘clinically significant’ decisional conflict appeared to be marginally higher in the standard compared with the enhanced group. The low average decisional conflict scores in the standard group may have made further substantial reductions unlikely [mean scores were 10.88 (SD 11.82) and 8.99 (SD 11.82) in the standard and enhanced groups respectively, with decisional conflict being scored on a scale of 0–100]. Of note, levels of decisional conflict in the PRIMETIME SWAT are similar to those in the IBIS II trial which investigated the use of a PDA in a randomised controlled trial of an aromatase inhibitor in patients at high risk of breast cancer (prevention group) and patients with DCIS (treatment group) [13.2 (SD = 14.5)] [14]. Most cancer clinical trials provide written information and do not usually include pictorial diagrams; incorporation of the diagrams was an intervention in itself and may have contributed to the low decisional conflict scores in the standard group. Furthermore, the SWAT may have been underpowered to detect a more modest effect size in terms of reduction in decisional conflict. However, with no guidelines available for defining clinically significant reduction in decisional conflict, the choice of statistical assumptions for designing this type of study has to be consensus led from the trialists.

### Study limitations

With respect to study limitations, only 36% of patients in the enhanced group reported having watched the video (data missing for 13%). The standard information may have been of sufficient quality to fulfil the information needs of these patients. Some patients may not have been made aware of the video or preferred not to have watched it at a potentially stressful time around their diagnosis. In addition, the SWAT was restricted to patients who were able to read and understand English independently in order to complete the questionnaire, although this is not an eligibility criteria restriction for the main study.

It is also possible that patients’ decisional conflict may have reduced over time irrespective of the video intervention as researchers at the centres became more experienced at discussing the trial with patients and other trial procedures—termed a ‘learning curve’. Furthermore, in a stepped-wedge trial where all centres begin in the control group, this learning curve would disproportionately adversely affect the control compared with the intervention group. A sensitivity analysis of the primary endpoint excluding patients who had returned questionnaires in the first two months of the sub-study being open in their centre was done, but this did not affect the results. Research teams at sites may have adapted the way they described the study after having watched the video themselves, although this was not measured. Implementing a new intervention mid-way through a trial may have been a challenge for sites and an alternative would have been to use a parallel-group cluster randomised trial design whereby clusters are allocated which type of information to use throughout the duration of the trial (albeit with the option of all clusters getting access to the intervention at the end of the study).

### Clinical implications

PDA were designed in collaboration with patients to enhance information for those considering treatment de-escalation. The SWAT concept enabled these to be tested in an efficient and economic manner. Levels of decisional conflict were low on average in patients receiving standard information incorporating diagrams. The diagrams alone may have resulted in low decisional conflict scores in the standard group, such that further substantial reductions would be unlikely. De-escalation trials can be a challenge to conduct and recruit to, albeit acceptance to PRIMETIME is high. In general, patients may perceive that ‘more is better’ and clinicians may practice ‘better safe than sorry’ [22]. This emphasises the importance of patient-led information delivery to ensure patients understand and feel comfortable with the trial especially in the era of treatment de-escalation.

## Conclusion

The low levels of decisional conflict in PRIMETIME are reassuring and may reflect the high-quality information provision including diagrams designed by patients for patients in collaboration with researchers, such that not everyone required the video. This reinforces the importance of working with patients as partners in clinical trials especially in the development of patient-centred information and decision aids. Furthermore, in an era of increasing use of virtual clinic appointments and consent, videos are an invaluable resource to help patients make informed decisions regarding breast radiotherapy.

## Supplementary Information

**Additional file 1: Appendix figure 1.** Questionnaire given to patients in the standard group*.* **Appendix figure 2.** Questionnaire given to patients in the enhanced group*.* **Appendix figure 3.** Summary of the risk of recurrence in very low risk patients. **Appendix figure 4.** Summary of change in breast appearance in women treated with radiotherapy. **Appendix table 1.** Summary of script structure. **Appendix table 2.** Summary of association of age and education level with decisional conflict. **Appendix table 3.** Summary of decisional conflict subscales results.

## Data Availability

The datasets used and/or analysed during the current study are available from the corresponding author on reasonable request.
