# Supplementary material for: Can patient decision aids reduce decisional conflict in a de-escalation of breast radiotherapy clinical trial? The PRIMETIME Study Within a Trial implemented using a cluster stepped-wedge trial design
Source: Trials. 2021 Jun 14;22:397. doi: 10.1186/s13063-021-05345-y (PMC8202048; doi:10.1186/s13063-021-05345-y)
Supplement: Supplementary file 1 — Additional file 1: Appendix figure 1. Questionnaire given to patients in the standard group. Appendix figure 2. Questionnaire given to patients in the enhanced group. Appendix figure 3. Summary of the risk of recurrence in very low risk patients. Appendix figure 4. Summary of change in breast appearance in women treated with radiotherapy. Appendix table 1. Summary of script structure. Appendix table 2. Summary of association of age and education level with decisional conflict. Appendix table 3. Summary of decisional conflict subscales results. [file 13063_2021_5345_MOESM1_ESM.docx]

**Appendices**

**Appendix figure 1:** Questionnaire given to patients in the standard group


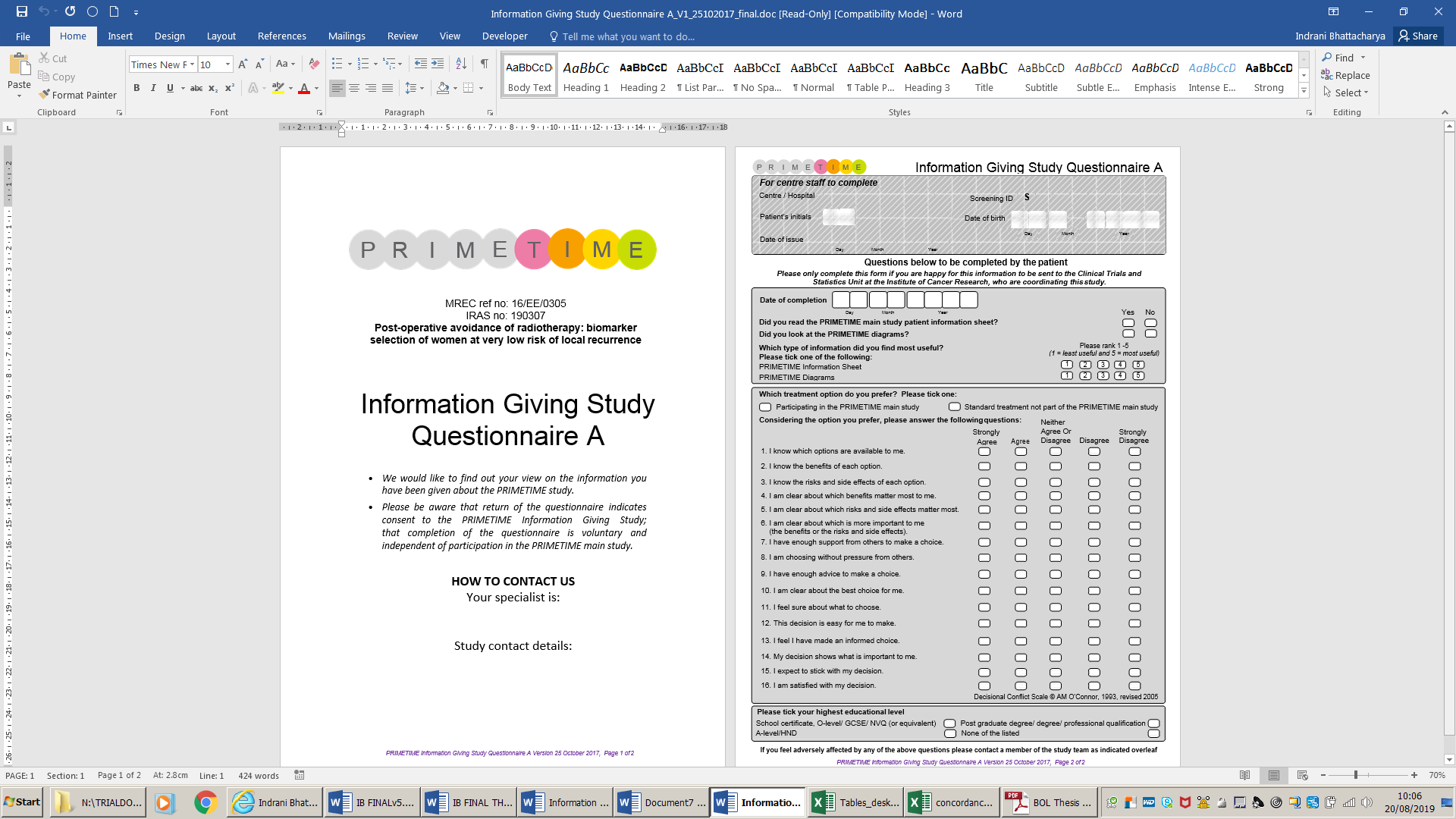


**Appendix figure 2:** Questionnaire given to patients in the enhanced group

**Appendix figure 3:** Summary of the risk of recurrence in very low risk patients


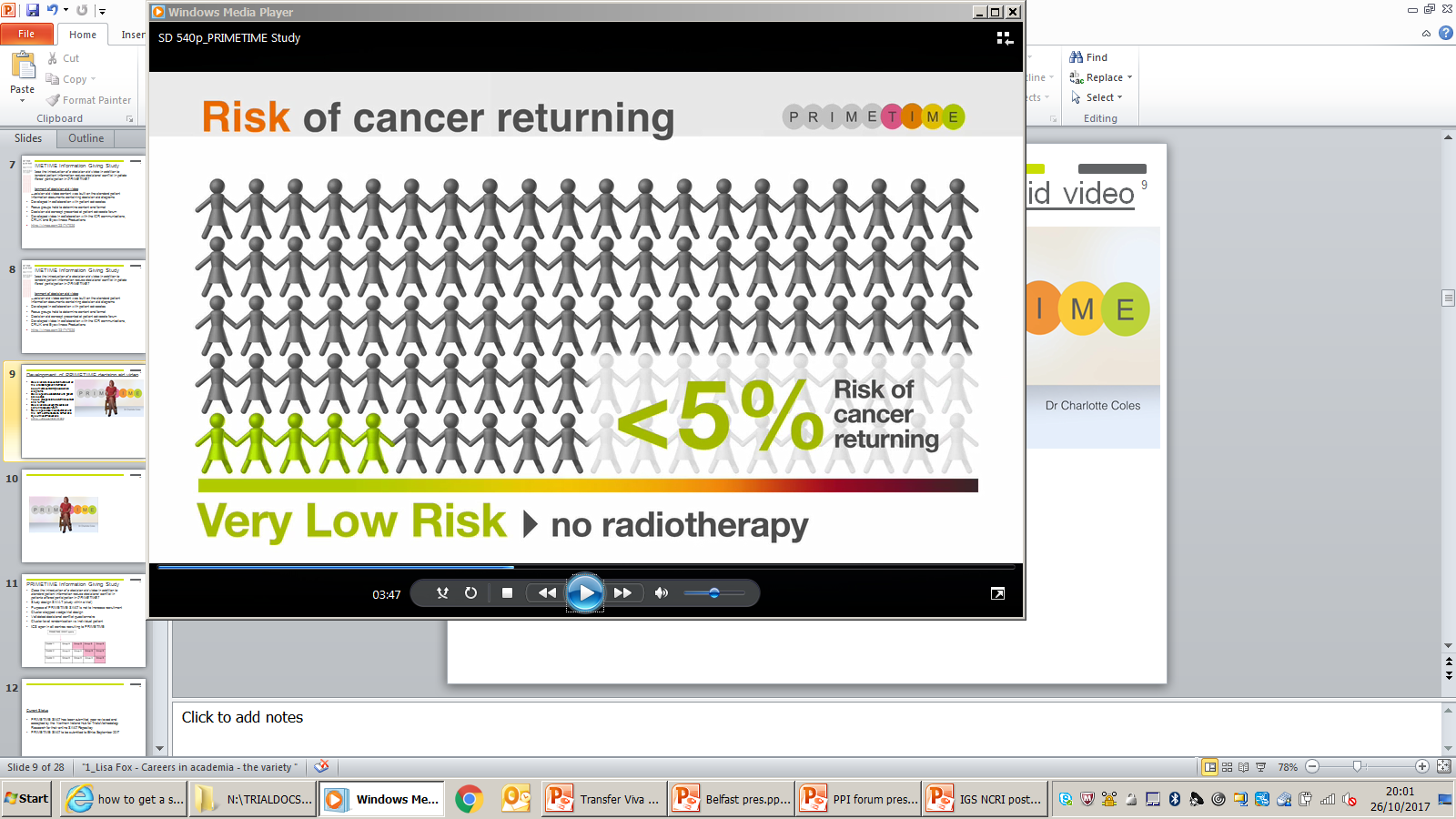


**Appendix figure 4:** Summary of change in breast appearance in women treated with radiotherapy


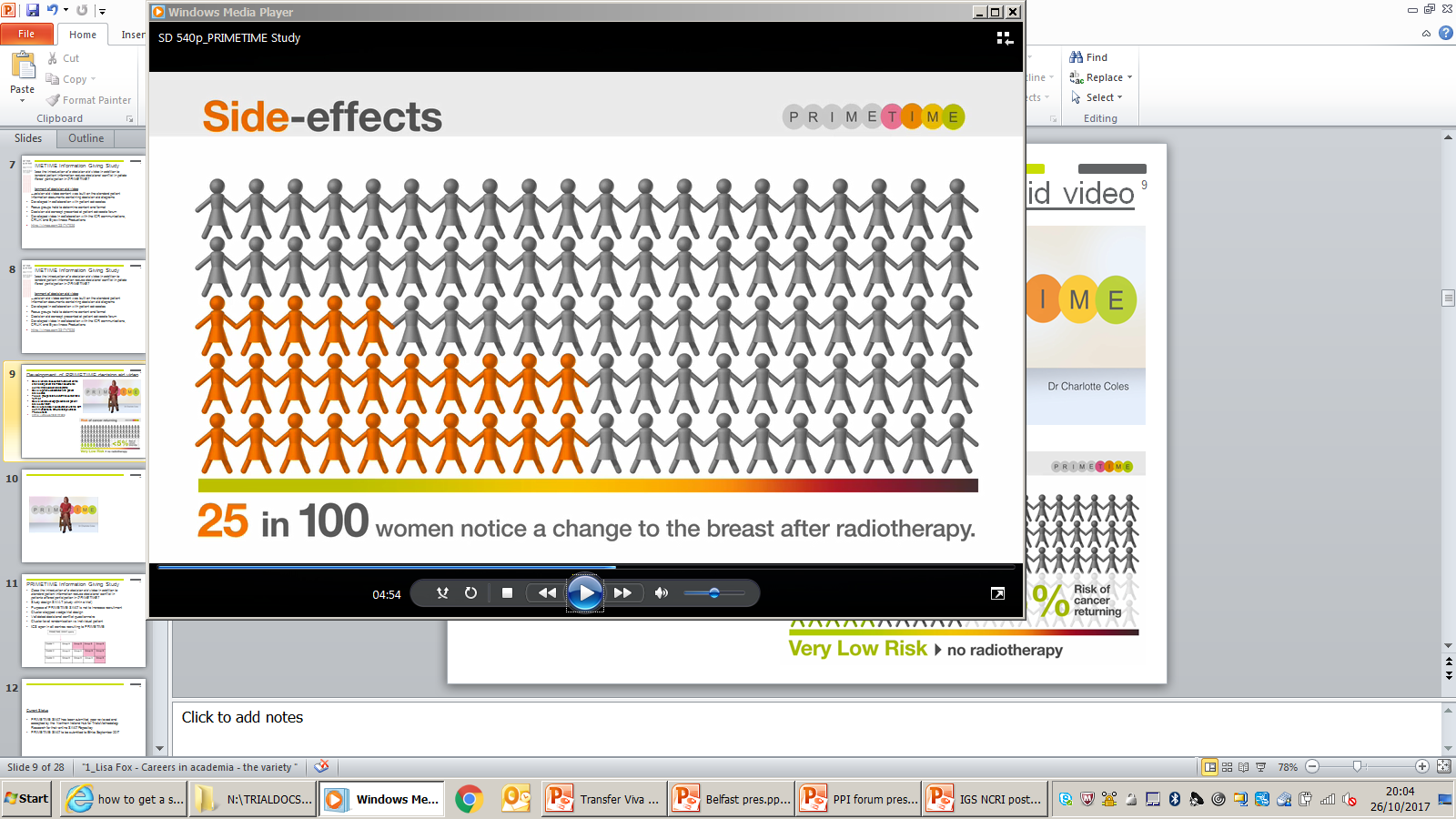


**Appendix table 1:** Summary of script structure

**Appendix table 2:** Summary of association of age and education level with decisional conflict

| **Baseline characteristics**  **(n= patients with available data)** | **Mean decisional conflict (SD)** | **Median decisional conflict (IQR)** | ***Coefficient value (95% confidence interval), p value** |
| --- | --- | --- | --- |
| Age (years)  60-64 (n=64)  65-69 (n=100)  70-74 (n=101)  ≥75 (n=53)  Age tested as a continuous variable | 9.58 (14.28)  9.22 (10.91)  8.69 (10.53)  12.59 (11.99)  - | 1.56 (0-17.19)  1.56 (0-20.31)  3.13 (0-17.19)  10.94 (0-25)  - | -  0.12 (-2.49-2.74), p=0.93  -0.69 (-3.65-2.28), p=0.65  3.67 (-0.47-7.82), p=0.08  0.12 (-0.08-0.31), p=0.25 |
| Education Level  PG degree/degree (n=75)  A-level/HND (n=49)  School cert/O-level (n=103)  Not listed (n=75)  Education tested as a continuous variable | 7.87 (12.80)  9.71 (10.69)  9.31 (11.21)  11.08 (12.31) | 1.56 (0-12.5)  4.69 (0-17.19)  3.13 (0-20.00)  4.69 (0-25) | -  1.79 (-2.51-6.10), p=0.41  1.57 (-1.84-4.97), p=0.37  2.89 (-0.63-6.41), p=0.11  0.86 (-0.27-1.99), p=0.22 |

*Coefficient value represents the difference between the means of the decisional conflict scales in the standard and enhanced groups

**Appendix table 3:** Summary of decisional conflict subscales results

| **Decisional Conflict Subscales** | **Mean score in Standard Group (standard deviation)**  **N=184** | **Mean score in Enhanced Group (standard deviation)**  **N=337** | ***Coefficient value (95% confidence interval)** | **P value** |
| --- | --- | --- | --- | --- |
| Uncertainty | 13.45 (15.82) | 11.98 (15.30) | -1.03 (-3.84-1.78) | 0.47 |
| Informed | 9.84 (11.78) | 7.90 (11.91) | -2.04 (-4.17-0.09) | 0.06 |
| Values Clarity | 11.93 (14.36) | 9.25 (13.35) | -2.46 (-4.85-0.06) | 0.05 |
| Support | 8.82 (11.78) | 7.12 (11.37) | -1.65 (-3.73-0.43) | 0.12 |
| Effective | 10.47 (12.96) | 8.78 (12.82) | -1.74 (-3.91-0.42) | 0.11 |

*Coefficient value represents the difference between the means of the decisional conflict scales in the standard and enhanced groups
